# Flexible Three-Dimensional Reconstruction via Structured-Light-Based Visual Positioning and Global Optimization

**DOI:** 10.3390/s19071583

**Published:** 2019-04-01

**Authors:** Lei Yin, Xiangjun Wang, Yubo Ni

**Affiliations:** 1 State Key Laboratory of Precision Measuring Technology and Instruments, Tianjin University, No. 92 Weijin Road, Nankai District, Tianjin 300072, China; zgkdxzylei@163.com; 2 MOEMS Education Ministry Key Laboratory, Tianjin University, No. 92 Weijin Road, Nankai District, Tianjin 300072, China; niyubo@tju.edu.cn; 3 Beijing Key Laboratory of Urban Spatial Information Engineering, No. 15 Yangfangdian Road, Haidian District, Beijing 100089, China

**Keywords:** 3D reconstruction, stereo vision, structured light, pose estimation, global optimization

## Abstract

Three-dimensional (3D) reconstruction using line structured light vision system commonly cooperates with motion restraint devices, such as parallel guide rail push-broom devices. In this study, we propose a visual positioning method to eliminate the motion constraint. An extended orthogonal iteration algorithm for visual positioning is proposed to obtain the precise position of the line structured light binocular camera system during movement. The algorithm uses the information acquired by the binocular camera, and produces a better positioning accuracy than the traditional vision localization algorithm. Furthermore, a global optimization method is proposed to calculate the poses of the camera relative to the world coordinate system at each shooting position. This algorithm effectively reduces the error accumulation and pose drift during visual positioning, and 3D information of the surface can be measured via the proposed free-moving line structured light vision system. The simulation and physical experiments performed herein validate the proposed method and demonstrate the significant improvement in the reconstruction accuracy: when the test distance is 1.5 m, the root mean square error of the point cloud is within 0.5 mm.

## 1. Introduction

Vision measurement is widely used in the field of industrial measurement [1,2,3,4,5]. Vision-based structured light measurement methods can effectively improve the precision of the measurement. Structured light is becoming increasingly popular in areas such as surface reconfiguration, vision navigation and workpiece inspection [6,7,8,9]. There are various forms of structured light, such as dot structured light, line structured light and surface structured light. Dot structured light has low surface reconstruction efficiency and is only suitable for several special scenarios. Surface structured light has various forms, such as grating phase-based sine stripe structured light and Gray code time series-based coding structured light. Surface structured light is projected by a digital light processing (DLP) projector. Due to the power limitation of the projector, the surface structured light is normally more suitable for high precision reconstruction of small indoor workpieces than for large surfaces and outdoor scenes. Line structured light has strong light intensity and can be projected to long distance; therefore, its application is very extensive. Line structured light typically requires a push-sweep motion when reconstructing a measured surface. The traditional structured light push-broom device mostly relies on a parallel guide rail. However, in many cases, it is not suitable to use the guide rail that limits the application of the line structured light. Therefore, to address these problems, we use the visual positioning algorithm to remove the restriction of the slideway, and proposed a surface reconstruction method based on binocular camera and structured light. This system projects line structured light on the measured surface via a high-power linear laser projector and obtains the motion trajectory through the visual positioning algorithm. Moreover, to obtain accurate pose information of the system during movement, an extended orthogonal iterative algorithm that fits the binocular camera system is proposed. In addition, we propose a global optimization algorithm to calculate the camera poses relative to the world coordinate system at each shooting position. Through the pose information of the system, the coordinates of the structured light stripes in the world coordinate system can be restored, and then the surface topography of the measured object can be obtained.

## 2. Related Work

Visual measurement technology can be divided into two categories: active methods and passive methods. Passive methods do not rely on illumination technology such as stereo vision measurement [10,11,12]. The stereo vision method reconstructs the surface topography of the measured object through photos taken from different perspectives, and the main process includes image pre-processing, feature point matching, and spatial point cloud coordinate calculation. However, when an object surface has insufficient texture information, stereo vision method cannot provide accurate reconstruction results. On the contrary, active vision methods mainly rely on structured light, such as line structured light and coding surface structured light [13,14]. The former is widely applied in industrial non-contact measurement scenarios such as weld seam inspection and rail wear detection. Wang et al. [15] proposed a method for measuring the contour of a track using line structured light. In contrast to the conventional measurement methods, this method used multi-line structured light along with the collinearity and parallelism constraints of the feature points on the laser plane to calibrate the structured light to avoid measurement errors caused by structural variation. In addition, the contour deformation error caused by system vibration was solved by projecting the light stripe to the track cross section plan. Li et al. [16] described a method for weld inspection using line structured light. In this study, a detailed vision measurement model was described, and a corresponding image processing method was provided for the arc reflection situation in the welding process. Interference points were removed using the space and time constraint algorithm, and the laser light stripes were extracted precisely by Gaussian filtering and linear interpolation. Li et al. [17] performed a study on the reconstruction of road ruts using a binocular structured light system. A structured light was calibrated using a two-dimensional target, and the road ruts were reconstructed by extracting the light stripes. To comprehensively evaluate the parameters of the road rut, the authors proposed a method for extracting the depth and area of the ruts using the rutting support point. Usamentiaga et al. [18] described a method for three-dimensional (3D) reconstruction using line structured light that was primarily applied in vibration scenes. The object profile information was reconstructed using the extracted multi-line structured light stripes to obtain the vibration pattern of the object. The vibration information was removed by mathematical modeling. In the experiment, the vibration removal effects of multi-line structured light and dual-line structured light were compared. The experiment showed that the dual-line structured light achieved better results than those of the multi-line structured light system. In addition, numerous studies have focused on the calibration method of line structured light. Xie et al. [19] introduced a structured light calibration method. In this paper intersection points between the laser plane and the grid line target were used as feature points to calculate the intrinsic and extrinsic parameters of the system. There were two categories of feature points. The first was the collinear feature points, and the coordinates of these points in the target coordinate system were calculated using the principle of cross-ratio invariance. The second category was the non-collinear points. Non-collinear points were obtained by multiple movements of the coordinate measuring machine, the intrinsic parameters and the extrinsic parameters of the system were solved by converting the coordinates of the feature points into the coordinate system of the coordinate measuring machine. Liu et al. [20] introduced a line structured light calibration method suitable for complex light environments. Two parallel cylinders with identical diameters were used for calibration. Line structured light was projected onto cylinders, and the light stripe images were captured. The elliptic equation of the light stripe in the image was obtained using the fitting method, and the relationship between the intersection line and the image eclipse was established using the perspective projection model. The optical plane equation was solved based on the constraint that the short axis of the ellipse is equal to the diameter of the cylinder.

Likewise, the application of coded structured light is also very extensive: for instance, structured light coding is used to mark the surface of the measured object [13]. After calibration, the depth information of the object surface can be calculated to reconstruct the surface of the object [21,22]. Color coded structured light uses the color information to mark the surface of the object. The advantage of this method is that the surface depth information of the object can be obtained from a single image. However, when the color or the reflection rate of the object interferes with the color of the structured light, the reconstruction precision gets affected. The sequence projection technique projects a series of patterns onto the surface of the object through a projector, including sinusoidal stripes, binary codes, and Gray codes [23,24,25]. The grayscale coding information about each point on the object surface is demodulated to obtain the corresponding projector pixel coordinates. Thus, the depth information is reconstructed. However, the discontinuous object surface or non-diffuse reflection surface reduces the measurement accuracy of the sine stripe method. The binary code and Gray code technologies are more reliable and insensitive to object surface characteristics. However, to obtain a high spatial resolution, several patterns should be projected, therefore, these methods are only suitable for static measurement scenes.

## 3. System Architecture and Optimization Algorithm

The proposed structured light surface reconstruction system consists of a binocular camera and a high-power line structured light projector. The surface of the measured object is reconstructed using a push-broom motion of light stripes on the measured surface. The binocular camera is used to recover the motion trajectory of the system and obtain structured light information. The traditional vision structured light systems mostly rely on a parallel guide rail. These systems are fixed on a parallel guide rail to scan the measured object. Since the moving velocity and moving direction of the guide rail are known, the motion information of the structured light system can be determined. Based on this motion information, the surface topography of the measured object can be obtained by transforming the coordinates of the extracted light stripes to the world coordinate system. Another form of the push broom system is that the vision structured light system is fixed, the measured object is driven by a guide rail and moves at a constant speed, and the surface topography can be obtained by splicing the light stripes based on the motion pattern of the measured object. As the above methods require a guide rail, there are significant constraints on the application scenarios that make them unsuitable for outdoor scenarios or large surface reconstructions. 

**Figure 1:**
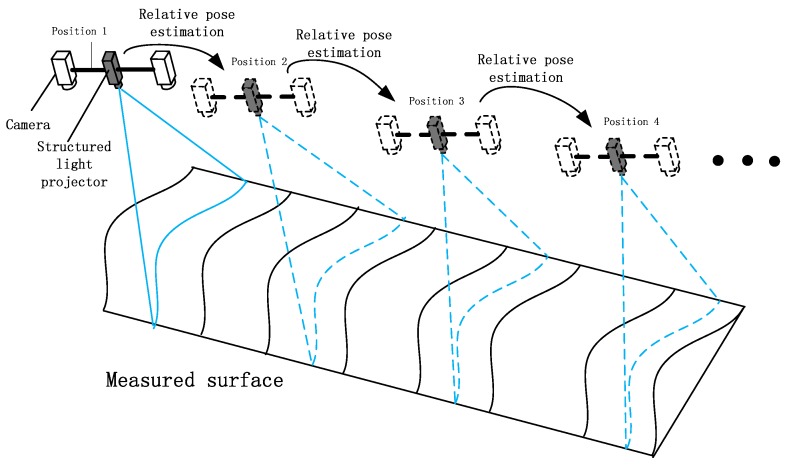
Schematic of surface reconstruction using push-broom structured light system.

To address these issues, this study proposes a structured light reconstruction system that moves freely without the restriction of a slideway. It adapts to various scenarios because of the high power of the line structured light. The system architecture is shown in Figure 1. While moving, the binocular camera captures the structured light stripe. At the same time, the pose information of the system in the world coordinates system is restored by the inter-frame match point track method, and then the motion trajectory of the system is obtained. The light stripes taken at various positions are restored to the world coordinate system by the motion pose parameters of the system; thus we can obtain the morphology of the curved surface. 

### 3.1. System Localization Method

The critical technology of the free-moving structured light push-broom system is the recovery of the system’s motion trajectory and pose information. The motion posture recovery precision directly affects the reconstruction precision. To obtain precise system posture information during movement, an extended orthogonal iterative algorithm is designed for the binocular system. The traditional orthogonal iterative algorithm [26] is only suitable for monocular cameras, whereas the proposed extended orthogonal iterative algorithm makes full use of the binocular information for pose calculations. The orthogonal iterative algorithms are primarily used for posture measurements of cooperative target. In the calculation process, it is necessary to know the coordinates of the feature points in the object coordinate system. In traditional algorithms, an artificial mark point must be posted. In the proposed algorithm, we use image feature points to replace artificial mark points, and the feature points are tracked. The 3D coordinates of the match points in the system coordinate system are calculated by the extrinsic parameters of binocular camera. The relative pose of the system between different positions is calculated by the extended orthogonal iterative algorithm through the tracking of the matching points set. To obtain the system pose relative to the world coordinate system at each shooting position, a global optimization algorithm has been designed. This algorithm effectively reduces the error accumulation in the calculation process and the motion trajectory drift problem. 

#### 3.1.1. Extended Binocular Orthogonal Iterative Algorithm

**Figure 2:**
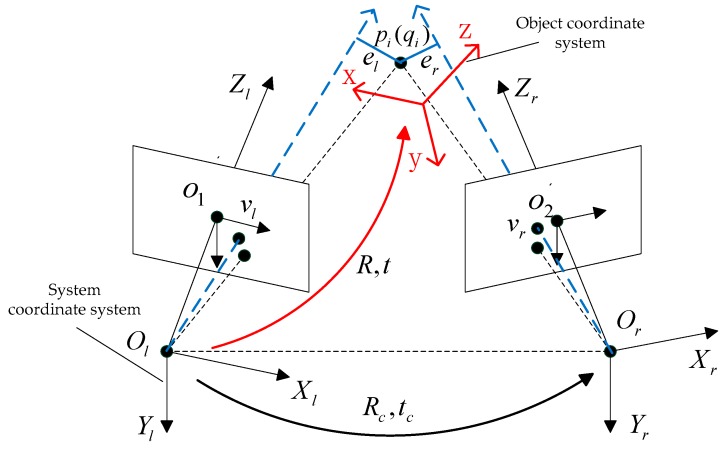
of the extended binocular orthogonal iterative algorithm.

The proposed extended orthogonal iterative algorithm is suitable for binocular systems. In contrast to the traditional orthogonal iterative algorithms, this algorithm can simultaneously use the feature points observed by both left and right cameras, which significantly increases the number of feature points in the calculation and effectively enhances the robustness of the algorithm. In the proposed extended binocular orthogonal iterative algorithm, the sum of the collinearity errors of the left and right camera is defined as the error function for the iteration. The principle of the collinearity errors of the two cameras is shown in Figure 2.

We use the camera coordinate system of the left camera as the system coordinate system, and the optical centre of the left camera is defined as the origin of the system coordinate system. Based on the orthogonal iterative algorithm [26], the spatial collinearity error of feature point $p_{i}$ observed by the left camera is defined as follows:

(1) $$e_{l}^{i}=(I-V_{l})(Rp_{i}+t)$$

where $V_{l}=v_{l}v_{l}^{T}/(v_{l}^{T}v_{l})$ is the projection matrix along the line of sight; $v_{l}$ denotes the normalized image coordinates of the feature point, $p_{i}$ denotes the coordinates of the feature point in the object coordinate system, *R* and *t* denote the rotational matrix and translation vector between the system coordinate system and the object coordinate system, respectively, and *I* denotes the identity matrix. 

To calculate the collinearity error of the right camera and maintain consistency with the collinearity error of the left camera, the coordinates of points observed by the right camera and the projection matrix along the line of sight of the right camera should be converted to the system coordinate system. By calibrating the extrinsic parameters, we can obtain the rotational matrix and translation vector of the right camera coordinate system relative to the left camera coordinate system. In Figure 2, $R_{c}$ and $t_{c}$ denote the rotational matrix and translation vector between the two cameras, respectively. In addition, we assume that the optical centre of the right camera is $O_{r}$ and that the coordinate of the feature point observed by the right camera in the system coordinate system is $q_{i}$. The vector $O_{r}q_{i}$ in the system coordinate system is expressed as follows:

(2) $$O_{r}q_{i}=Rp_{i}+t-t_{c}$$

if **v**_r_ denotes the image point coordinates of spatial point $p_{i}$ projected to the normalized phase plane of the right camera, then the coordinate of the vector $O_{r}v_{r}$ in the system coordinate system is expressed as follows:

(3) $$\begin{array}{l} O_{r}v_{r}=(R_{c}v_{r}+t_{c})-t_{c}\\ \;=R_{c}v_{r} \end{array}$$

The projection matrix of the right camera along the line of sight in the system coordinate system is expressed as follows:

(4) $$V_{r}=\frac{(O_{r}v_{r}){\left(O_{r}v_{r}\right)}^{T}}{{\left(O_{r}v_{r}\right)}^{T}(O_{r}v_{r})}=\frac{R_{c}v_{r}v_{r}^{T}R_{c}^{T}}{v_{r}^{T}R_{c}^{T}R_{c}v_{r}}$$

The objective function of the spatial collinearity error of point $p_{i}$ observed by the right camera is expressed as follows:

(5) $$e_{r}^{i}=(I-V_{r})(Rp_{i}+t-t_{c})$$

When *n*_1_ feature points are observed by the left camera and *n*_2_ feature points are observed by the right camera, the objective function of the extended orthogonal iterative algorithm is represented by the following expression:

(6) $$E(R,t)=\min(\sum_{i=1}^{n_{1}}{\left\|e_{l}^{i}\right\|}^{2}+\sum_{i=1}^{n_{2}}{\left\|e_{r}^{i}\right\|}^{2}),\;\mathrm{subject}\;\mathrm{to}\;R^{T}R=I$$

After the vector parameters in the coordinate system of the right camera are converted to the system coordinate system, the collinearity error function of the right camera $e_{r}$ and that of the left camera $e_{l}$ have identical forms. Then we can use the method in [26] to solve the above equation. Referring to the solution process of the orthogonal iterative algorithm, when the objective function is minimum, the *R* matrix and the *t* vector are the system pose relative to the object coordinate system.

#### 3.1.2. Calculation Process of Localization Algorithm

During the system positioning process, the image match points are used as mark points for pose estimation. The positioning process can be described as follows:

Step 1: The ORB features [27] of the binocular camera’s left and right images at the current position are extracted and matched.
Step 2: The mismatches are removed through the RANSAC algorithm [28].
Step 3: The 3D coordinates of the feature points at the current position are calculated from the extrinsic parameters of the binocular camera by the triangular method, and then these coordinates are saved to generate 3D point set.
Step 4: The ORB feature points of the image captured at the next position (or another position) corresponding to the current 3D point set are extracted by feature point tracking method.
Step 5: Based on the current 3D point set and the pixel coordinates of the matching points on the left and right images of the next position (or another position),the relative pose of the current and the next position (or another position) is calculated by the extended orthogonal iterative algorithm presented in Section 3.1.1.
Step 6: Steps 1 to 5 are repeated to obtain the relative pose of the system at each adjacent positions. When we define the system coordinate system at the first position as the world coordinate system, the system poses at each position relative to the world coordinate system can be obtained from the relative pose of the system between each adjacent positions.

### 3.2. Light Stripe Extraction and Splicing Method

The structured light stripes are extracted using the Steger algorithm [29,30]. The Steger method can extract sub-pixel information of structured light stripes. Before light stripe extraction, an epipolar rectification of the left and right images is performed by the Fusiello algorithm [31]. After the epipolar rectification, the vertical coordinates of the corresponding light stripe points in the left and right images become consistent, the corresponding points of the light stripes on the left and right images are conveniently determined, and the spatial 3D coordinates of the points on the strips are calculated using the triangulation method.

After obtaining the light stripe point cloud at each position, the light stripes are restored to the world coordinate system based on the system pose information at each position to reconstruct the surface topography. The pose information of the binocular structured light system at various positions is calculated using the method presented in Section 3.1.

*R* and *t* denote the calculated rotational matrix and translation vector of the system coordinate system relative to the world coordinate system at various positions, respectively. The coordinates of the light stripe point in the world coordinate system are represented by $P_{i}$, and the coordinates of the light stripe point in the system coordinate system calculated by the triangular method are represented by $P_{j}$, thus, the following relationship is established:

(7) $$P_{i}=R^{-1}P_{j}-t$$

From the above formula, the coordinates of the light stripe point in the world coordinate system can be obtained, and then the point cloud of the measured surface can be obtained.

The intrinsic parameters and the extrinsic parameters should be calibrated before the calculation process. We use the checkerboard target to calibrate the cameras, and the Calibration Toolbox for MATLAB [32] is used to calibrate these parameters. Since many parameters are involved in the calculation process, we summarize the pre-calibration parameters and the unknown parameters in Table 1.

### 3.3. Global Optimization Algorithm of Pose Estimation

The relative pose of the system between two adjacent positions is calculated by the method presented in Section 3.1.2. The system coordinate system at the first position is regarded as the world coordinate system. According to the relative pose of the system between adjacent positions, the poses of the system relative to the world coordinate system at each shooting position can be calculated one by one. This pose calculation method is widely used in incremental 3D reconstruction. In this method, the pose of the system in the world coordinate system depends on the accuracy of the pose at the previous position; however, this could result in an error accumulation. The global optimization algorithm effectively prevents this problem. The idea of global optimization algorithm is to combine all associated frames based on match points and calculate the pose relative to the word coordinate system in a unified framework that can effectively suppress the cumulative errors caused by the incremental reconstruction method and significantly reduce the system positioning error in the world coordinate system at each position.

#### 3.3.1. Global Optimization Method of Rotation Matrices

To obtain accurate pose calculation results, the rotational matrix and translation vector are optimized separately. The idea of global optimization of the rotational matrix is inspired by reference [33]. We assume that the camera’s rotational matrix at position i relative to the world coordinate system is $R_{i}$. The camera’s relative rotational matrix between positions i and j is $R_{ij}$. When the images taken at position *i* and position *j* have a certain number of matching points, the rotation matrix $R_{ij}$ of the system between the two positions can be calculated by the extended binocular orthogonal iterative algorithm explained in Section 3.1.1. We can get the following formula:

(8) $$R_{j}=R_{ij}R_{i}$$

Equation (8) can be divided into three parts:

(9) $$\begin{array}{l} r_{1}^{j}-R_{ij}r_{1}^{i}=0_{3\times 1}\\ r_{2}^{j}-R_{ij}r_{2}^{i}=0_{3\times 1}\\ r_{3}^{j}-R_{ij}r_{3}^{i}=0_{3\times 1} \end{array}$$

where $r_{1}^{i}$, $r_{2}^{i}$ and $r_{3}^{i}$ denote three columns of the matrix $R_{i}$, i.e., $R_{i}=\left[\begin{array}{ccc} r_{1}^{i} & r_{2}^{i} & r_{3}^{i} \end{array}\right]$. Equation (9) can be rearranged to the following linear equation group:

(10) $$\left[\begin{array}{cccccc} -R & 0 & 0 & I & 0 & 0\\ 0 & -R & 0 & 0 & I & 0\\ 0 & 0 & -R & 0 & 0 & I \end{array}\right]\cdot \left[\begin{array}{c} r_{1}^{i}\\ r_{2}^{i}\\ r_{3}^{i}\\ r_{1}^{j}\\ r_{2}^{j}\\ r_{3}^{j} \end{array}\right]=\left[\begin{array}{c} 0_{3\times 1}\\ 0_{3\times 1}\\ 0_{3\times 1} \end{array}\right]$$

When the relative pose $R_{ij}$ between position *i* and position *j* is obtained by using the extended orthogonal iteration algorithm, the rotation $R_{ij}$, $R_{i}$, $R_{j}$ can be written in the form of Equation (10). These equations can be combined and written into an over-determined linear equation group in the form of Ax=0, where x is the combination of the three columns in the rotational matrix of the system relative to the world coordinate system, and A is composed of the relative rotational matrix $R_{ij}$ and the identity matrix $I_{3\times 3}$. By solving the linear equation, the system rotation matrix $R_{i}$ relative to the world coordinate system can be calculated by the least squares method. To ensure the orthogonality of the rotation matrix $R_{i}$, the orthonormality constraints are forced by projecting the approximate rotation to the closest rotation in the Frobenius norm using SVD [34]. Let the singular value decomps of R be USV^T^, and then the closest orthogonal matrix in Frobenius norm is R’ = UV^T^. We can get the accurate rotation matrix by replacing R with R’. In this way, the rotation matrices of the system at each position relative to the world coordinate system are solved by the relative rotation matrices of all associated positions. This method effectively utilizes redundant pose information, reduces the accumulation of measurement noise, and improves the positioning accuracy. In this study, we use a linear method to solve the equation and then use SVD to guarantee the orthogonality of the rotation matrix. This method has high computational efficiency and is suitable for situations with limited computational resources. When the computing resources are sufficient, the Lagrangian multiplier method can be used to calculate the equations with orthogonal constraint as a penalty factor.

#### 3.3.2. Global Optimization Method of Translation Vectors

Based on the rotational matrix of the system relative to the world coordinate system, the translation vector optimization method is introduced. Assuming that the system translation vector at position i relative to the world coordinate system is $c_{i}$ and the relative translation vector between positions i and j is $t_{ij}$. Then, we have the following expression [35]:

(11) $$R_{j}(c_{i}-c_{j})=t_{ij}$$

(12) $$i.e.,\;c_{i}-c_{j}=R_{j}^{-1}t_{ij}$$

Equation (12) can be rearranged to the linear equation group:

(13) $$\left[I_{3\times 3}{\;|\;-I}_{3\times 3}\right]\left[\begin{array}{c} c_{i}^{1}\\ c_{i}^{2}\\ c_{i}^{3}\\ c_{j}^{1}\\ c_{j}^{2}\\ c_{j}^{3} \end{array}\right]=\left[\begin{array}{c} t_{1}\\ t_{2}\\ t_{3} \end{array}\right]$$

where $c_{i}={\left[\begin{array}{ccc} c_{i}^{1} & c_{i}^{2} & c_{i}^{3} \end{array}\right]}^{T}$, $c_{j}={\left[\begin{array}{ccc} c_{j}^{1} & c_{j}^{2} & c_{j}^{3} \end{array}\right]}^{T}$, and $R_{j}^{-1}t_{ij}={\left[\begin{array}{ccc} t_{1} & t_{2} & t_{3} \end{array}\right]}^{T}$. When the image taken at position *i* and position *j* has a certain number of matching points, the relative translation vector of the system between the two positions can be calculated by the extended binocular orthogonal iterative algorithm in Section 3.1.1. The translation vector can be rearranged into the form of Equation (13). All the equations can be combined to obtain an over-determined group of linear equations in the form of Ax=b, where x is made by stacking all the translation parameters $c_{i}$, A is a matrix consisting of the identity matrices $I_{3\times 3}$ and $-I_{3\times 3}$, and b is a column vector consisting of the elements in $R_{j}^{-1}t_{ij}$. Using the least squares method to solve the above equations, we obtain the translation vector $c_{i}$ of the system relative to the world coordinate system at each position. Thus, the translation vector of the system at each position relative to the world coordinate system is solved by the relative translation vectors of all associated positions; this effectively utilizes the redundant translation vector information and significantly suppresses error accumulation.

After $R_{i}$ and $c_{i}$ are calculated as described above, the pose matrix $\left[\begin{array}{cc} R_{i} & c_{i}\\ 0 & 1 \end{array}\right]$ of the system at each position relative to the world coordinate system can be obtained. When all the pose matrices are obtained, we use the results as the initial values and use the bundle adjustment to optimize all the poses. Using the algorithm described in Section 3.2, the coordinates of the stripes in the world coordinate system can be recovered and the surface topography can be reconstructed.

## 4. Experiment Results

### 4.1. Simulation Experiment of the Extended Binocular Orthogonal Iterative Algorithm

**Figure 3:**
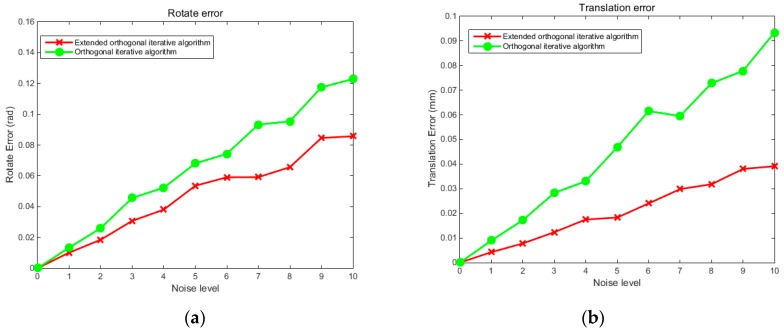
error curve of the rotation angle and translation affected by noise. Graph (**a**) shows the RMS error curve of rotation angle at different noise levels, and graph (**b**) shows the RMS error curve of translation at different noise levels.

**Figure 4:**
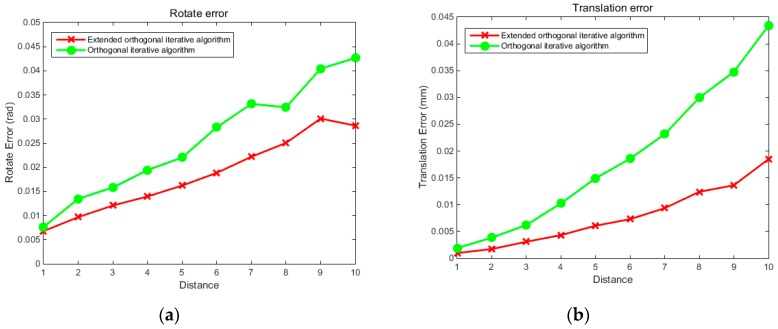
RMS error curve of rotation angle and translation affected by distance. Graph (**a**) shows the RMS error curve of the rotation angle at different distances, and graph (**b**) shows the RMS error curve of the translation at different distances.

Both simulation and physical experiments were carried out to test the proposed reconstruction method. First, to verify the stability and robustness of the localization method for the free-moving push-broom surface reconstruction system, we design a simulation experiment to compare the precision of the proposed extended binocular orthogonal iterative algorithm with that of the traditional orthogonal iterative algorithm, among which the traditional orthogonal iterative algorithm was implemented by using 3D points and their projections on the left camera. The two cameras used in the simulation experiment have the same intrinsic parameters, the focal length is set at 16mm, the image resolution is 1280 × 1024 pixel, the pixel size is 4.8 μm × 4.8 μm, and the principal point is at the image centre. By referring to the simulation model of the orthogonal iterative algorithm [25], we uniformly select 64 points in a space of [0, 15] × [0, 15] × [0, 15] × [0, 15] making up the feature point set of the target. The feature points are divided into two groups that are projected onto the image planes of the two cameras respectively to generate imaging points, with 48 points in each group. The extended orthogonal iteration algorithm can use imaging information of both left and right cameras, while the traditional orthogonal iteration algorithm can only use imaging information of the left camera. Ten levels of Gaussian noise with variance from 0 to 2 pixels were added to all the imaging points. In the experiment, the three rotation angles α, β and γ forming rotation matrix *R* were randomly generated from a uniform distribution. Five hundred tests were conducted at each noise level. The accuracy of the rotation was evaluated using the root mean square error (RMS) of three Euler angles:

(14) $$R\_\mathrm{noise}=\sqrt{\frac{1}{N}\sum_{i=1}^{N}\left({\left(\alpha^{\prime }-\alpha \right)}^{2}+{\left(\beta^{\prime }-\beta \right)}^{2}+{\left(\gamma^{\prime }-\gamma \right)}^{2}\right)}$$

where N denotes the number of test, $\alpha^{\prime },\beta^{\prime },\gamma^{\prime }$ denote the calculation results, and α,β,γ denote the true values. Figure 3 compares the accuracy of the proposed localization algorithm and the traditional orthogonal iteration algorithm at 10 levels of image noise. Figure 4 compares the accuracy of the two algorithms at different distances under an image noise level of 0.2 pixels. The abscissa in Figure 4 represents the ratio of the distance between the camera and the target to the size of the target: tz/16.

The experimental results shown in Figure 3 indicate that at each noise level, the proposed extended orthogonal iteration algorithm is superior to traditional orthogonal iteration algorithm in both rotational and translation accuracy. For instance, at the noise level of 2 pixels, the RMS rotation error of the traditional orthogonal iteration algorithm is 0.12 rad, while the RMS rotation error of the extended orthogonal iteration algorithm is less than 0.08 rad. The translation error of the traditional orthogonal iteration algorithm is 0.09 mm, while the translation error of the extended orthogonal iteration algorithm is less than 0.03 mm. Moreover, with an increase in image noise, the rotation and translation errors of the extended orthogonal iteration algorithm are significantly lower than those of the traditional method. It can also be seen from the error curves of the two algorithms shown in Figure 4 that the errors tend to increase as the distance increased, while the error corresponding to the extended orthogonal iteration algorithm is obviously reduced compared with that of the traditional algorithm. The above experimental results indicate that, since the available information for binocular system is more than that for monocular system, the proposed binocular localization algorithm is superior to the traditional orthogonal iteration algorithm under the same noise level. With an increase in distance, the positioning accuracy decreases gradually, but the positioning accuracy of the extended orthogonal iteration algorithm is higher than that of monocular orthogonal iteration algorithm.

### 4.2. Simulation Experiment of Global Optimization Algorithm

**Figure 5:**
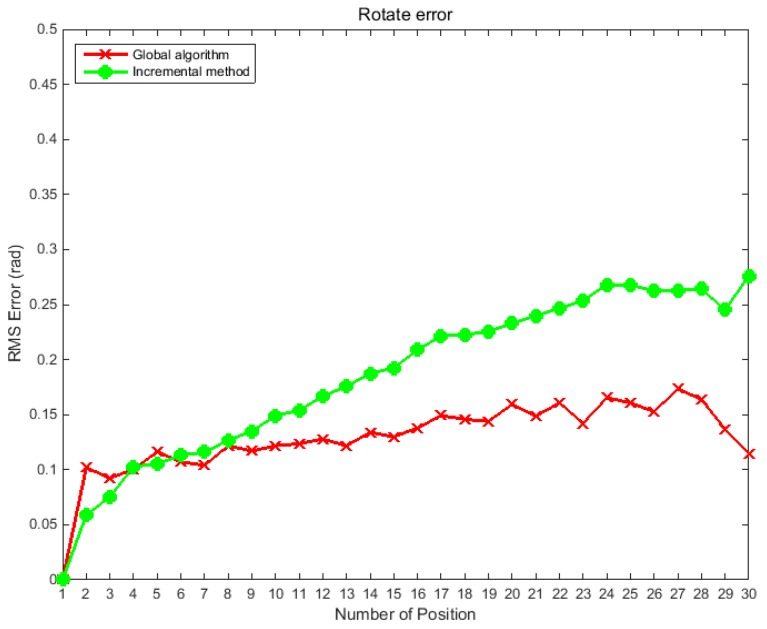
RMS error curves of rotation angle at different positions before and after global optimization.

A typical feature of the traditional incremental positioning method is that the pose of the camera in the world coordinate system at different positions depends on the pose accuracy of the previous camera, which easily causes an accumulation of errors. However, the global optimization algorithm effectively resolves this problem. To verify the reliability of the global optimization algorithm proposed in this study, a simulation experiment was performed. During the test, the binocular camera was made to perform a push-broom action; a total of 30 positions were moved, and the relative poses between adjacent positions were known values. Both the proposed algorithm and the incremental algorithm were used to calculate the pose of the camera at each position relative to the world coordinate system. After calculation, both methods were optimized by bundle adjustment. To further test the robustness of the algorithm, Gaussian noise was added to the relative pose of the adjacent position. In the first simulation experiment, a Gaussian noise with a variance of 0.1° was added to the three Euler angles of the relative pose between any two adjacent positions. The global optimization algorithm was compared with the incremental algorithm. The experiments were performed 500 times, and the RMS error of the two methods at different positions was calculated; the evaluation method for rotation was the same as that used in Section 4.1. The simulation results are shown in Figure 5. 

**Figure 6:**
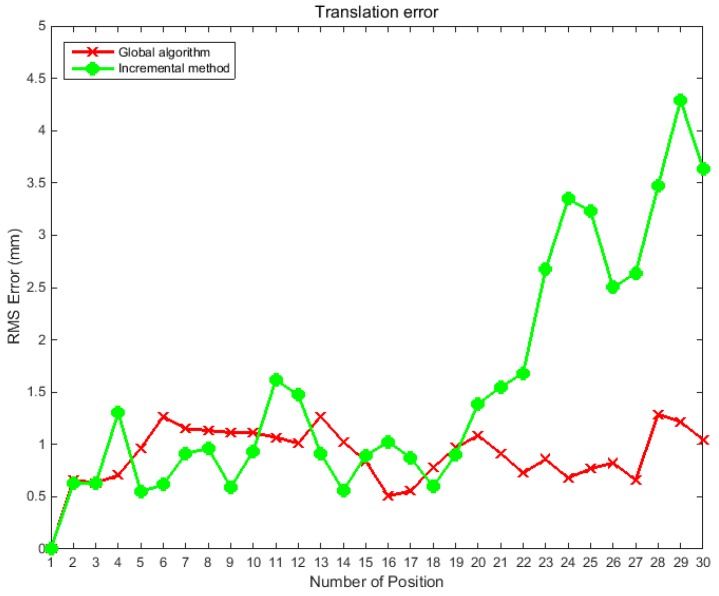
RMS error curves of translation at different positions before and after global optimization.

In the second simulation experiment, Gaussian noise with a variance of 5 mm was added to the three translations of the relative pose between any two adjacent positions to verify the robustness of the global optimization algorithm. The results produced by the global optimization algorithm were compared with those of the incremental algorithm. The experiments are performed 500 times, Figure 6 shows the RMS error of the calculated values and the true values of the camera pose in the world coordinate system at different positions.

The above simulation experiments show that the incremental method often results in large drifting error. As the push-broom motion progresses, the error of rotation and translation gradually increases. The global optimization algorithm effectively avoids error transmission and accumulation. Figure 5 shows that under the interference of noise, the angle error of the incremental method at the 30th position reaches 0.28 rad, while the error of the global optimization method is always within 0.15 rad. Similarly, Figure 6 shows that the errors of the incremental method are transmitted step by step; and while the location error of the translation at the 30th position using the incremental method is 4.3 mm, the location error of translation using global optimization algorithm is always within 1.5 mm.

### 4.3. Free-Form Surface Reconstruction Experiment

**Figure 7:**
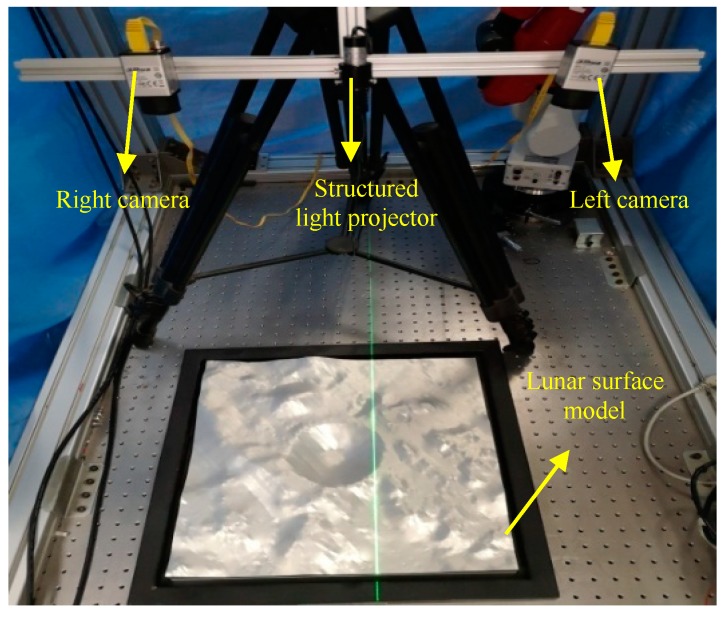
The schematic diagram of the experimental device.

To verify the correctness of the above methods and processes, we used a high-precision lunar surface model. The experimental setup is shown in Figure 7. 

The size of the lunar model used in the experiment is 0.5 m × 0.5 m. The image resolution of the cameras used in the experiment is 1280 × 1024 pixels, the pixels size is 4.8 μm × 4.8 μm, and the focal length is 12 mm. The laser wavelength is 532 nm, the power is 150 mW, and the contour width is less than 1 mm when the visual distance is 2 m.

**Figure 8:**
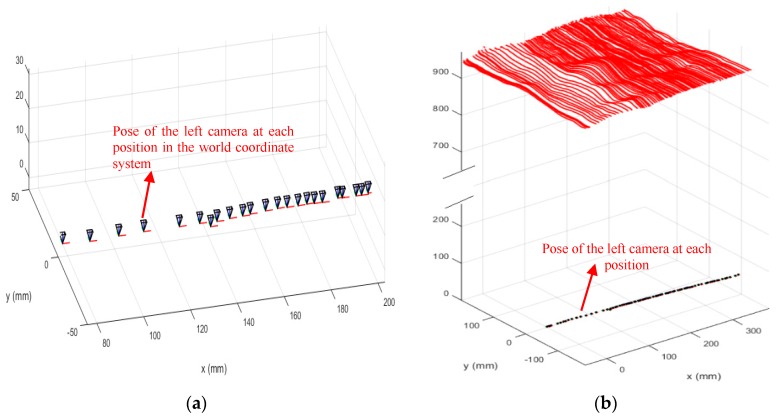
Splicing the light strips based on the camera pose information. Graph (**a**) shows the pose information of the left camera at the first 23 positions, and graph (**b**) shows positions of the restored light stripes and pose of the left camera in the world coordinate system.

**Figure 9:**
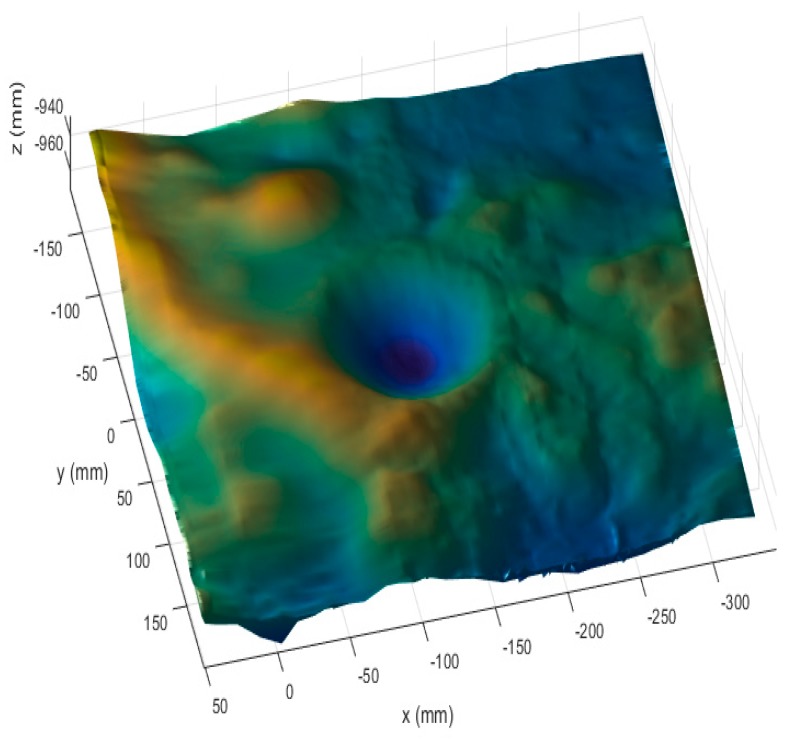
The rendering of the surface topography after triangulation of the point cloud and surface fitting.

First, the binocular system was calibrated, and the calibration results are presented in Table 2. The structured light stripes were projected onto the model while the binocular structured light system performed a push-broom motion. The binocular camera captures images in real time. Using the system coordinate system at the first position as the world coordinate system, a total of 78 images were taken during the movement. The extended binocular orthogonal iteration algorithm was used to recover the pose information of the system at the adjacent positions, and the global optimization of the pose was performed according to the algorithm described in Section 3.3. Thus the position of the system in the world coordinate system at each position was obtained. In the world coordinate system, the pose information of the left camera at the first 23 positions is shown in Figure 8a. The recovered camera pose was used to restore the position of the light stripes according to the method described in Section 3.2. The positions of the restored light stripes and left camera pose in the world coordinate system are shown in Figure 8b, while Figure 9 is a rendering of the surface topography after triangulation of the point cloud and surface fitting.

Figure 8b shows that the restored light stripes are clear and continuous, and the average spacing between the 3D points is 4.9 mm. On average, there are 73,582 3D points on the entire surface and 943 3D points on a single light stripe. From the observations made by rotating and scaling the reconstructed surface, the reconstructed results were found to be in good agreement with the details of the terrain surface. Among the more than 70,000 3D points, the number of gross-error points outside the entire surface was very small and the reconstruction accuracy was high. In the next section, we used a flat calibration board to quantitatively evaluate the reconstruction accuracy.

### 4.4. Evaluation Experiment of Reconstruction Accuracy

**Figure 10:**
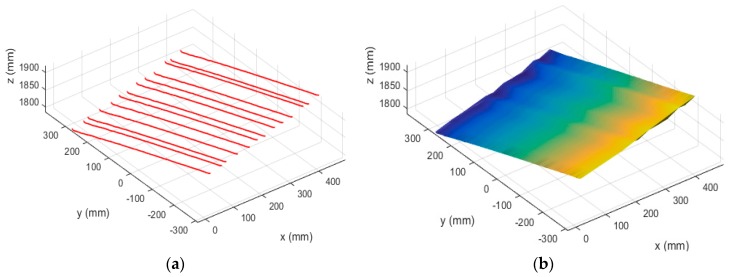
Reconstruction results of the flat calibration board before global optimization. Graph (**a**) shows the recovered positions of the light stripes in world coordinate system, and graph (**b**) shows the surface after triangulation and surface fitting.

In the experiment described in Section 4.3, we have verified the correctness of the proposed algorithm and procedure by performing a reconstruction of a free-form surface model. In this section, we designed an experiment to evaluate the reconstruction accuracy wherein a flat calibration board was reconstructed, and the reconstruction precision was evaluated by the flatness of the 3D points on the light stripes. The structured light system used in this experiment was the same as that used in the experiment in Section 4.3. The size of the flat calibration board is 60 cm × 60 cm, and the system performed a free push-broom movement at a distance of 1.5 m from the flat panel. During the experiment, 13 pictures of the structured light were taken from the moving system, and the stereo vision positioning method was used to recover the pose of the system in the world coordinate system; accordingly the positions of the light stripes were obtained. Both the incremental method and the global optimization method were used to recover the system pose. Figure 10 demonstrates the reconstructed surface of the calibration board without using the global optimization method: Figure 10a shows the recovered positions of the light stripes in world coordinate system, and Figure 10b shows the surface after triangulation and surface fitting. 

**Figure 11:**
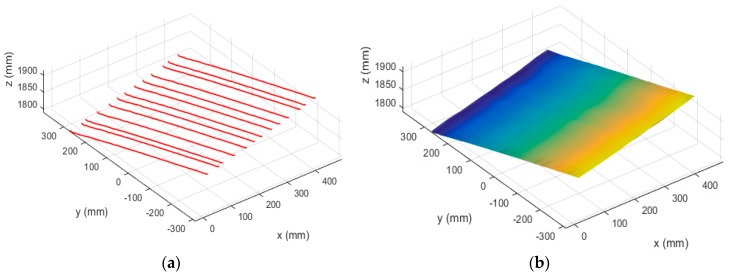
Reconstruction results of the flat calibration board after global optimization. Graph (**a**) shows the recovered positions of the light stripes in world coordinate system, and graph (**b**) shows the surface after triangulation and surface fitting.

Figure 11 demonstrates the reconstructed surface of the board using the global optimization method: Figure 11a shows the recovered positions of the light stripes in the world coordinate system, and Figure 11b shows the plane model after triangulation and surface fitting. Figure 10 and Figure 11 show that before using the global optimization algorithm the surface has minor fluctuations, while the surface after global optimization is smoother and shows better flatness.

We also quantified the magnitude of the flatness using the 3D points on the light stripes. A total of 9942 3D points were generated from the scanning, with an average of 764 3D points per light strip. We utilized 9931 points to fit the board plane, and calculated the distance from each point to the fitted plane. The calculation results (see Table 3) which reflected flatness were used as the basis to evaluate the reconstruction and optimization precision.

Table 3 indicates that the maximum and average distances between the 3D points to the fitted plane before using the global optimization algorithm are 2.32 mm and 0.76 mm, respectively, and the RMS error is 0.9052 mm. In contrast, the maximum and average distances between the 3D points to the fitted plane according to the data after using global optimization algorithm are 1.51 mm and 0.43 mm, respectively, and the RMS error is 0.4177 mm. The data show that the reconstruction accuracy of the plane is significantly improved by utilizing the global optimization algorithm.

**Figure 12:**
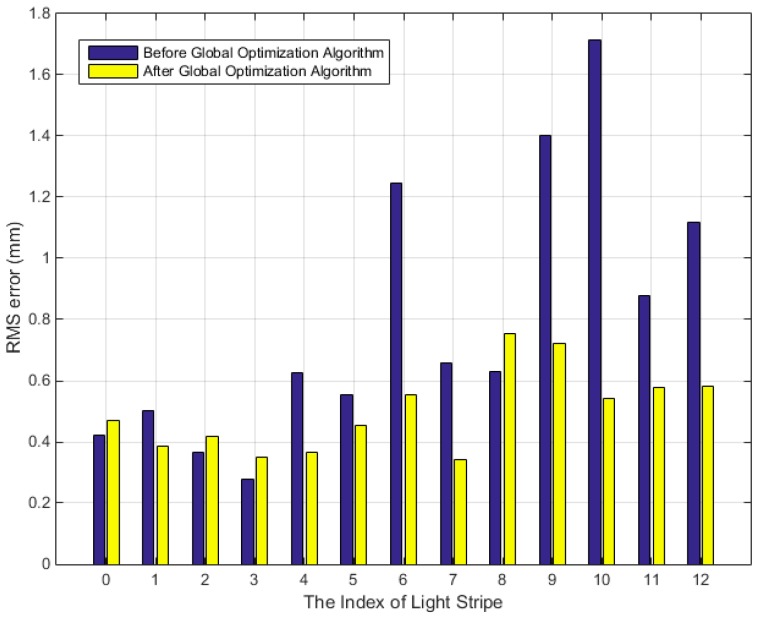
RMS errors of the distances between 3D points on light-stripes and the fitted plane before and after global optimization.

The RMS errors of the distances between the 3D points on 13 light stripes and the fitted plane before and after global optimization are shown in Figure 12. The RMS errors before optimization are observed to be higher and tend to increase with the increase in the number of light stripes. This result is in agreement with the explanation that the posture error accumulates gradually before global optimization. Furthermore, the precision is clearly observed to improve after global optimization, as the error of all light stripes is less than 0.5 mm. Since the measurement distance is 1.5 m, the relative error is 0.03%. The above experimental results show that the proposed algorithm is accurate and that the optimization method significantly improves the measurement results.

### 4.5. Discussion

The characteristic of this method is that it enables the system move freely. In this study a visual positioning method is proposed to eliminate the motion constraint. The motion trajectory is accurately restored by various optimization methods. It should be noted that when the number of pictures to be processed is large, the dimension of matrix A in Section 3.3.1 is large, which may result in a decrease in computational efficiency. The dimension of matrix A is relative to the number of images and the number of relative poses obtained from these images. Assuming a total of n pictures were taken, we obtained m relative rotation matrices in the n pictures using the method in Section 3.3.1, then the dimension of the A matrix is 9m × 9n. However, we can see that matrix A is sparse, so we use the Eigs function in MATLAB to calculate the linear equation by referring to the method in [33,34]. MATLAB’ Eigs function is suitable for solving sparse matrices with large dimension, and can solve the problem to some extent. In the experiment, we run all codes in MATLAB 2014b on a desktop with an i7 4790 CPU and 6 GB RAM, the number of photos was less than 100, and the calculation efficiency was not significantly reduced.

To demonstrate the advantages of this method more clearly, a comparative analysis is conducted with the existing method. Wang et al. [1] used standard industrial 3D scanners called TRITOP for three-dimensional reconstruction. Since the measurement range of the scanner is small, some artificial marked points are attached on measured object to expand the scanning range. In this study, at a measurement distance of 300 mm, the error is 0.05 mm, and the relative error is 0.016%. Yang et al. [25] used a binocular system to capture the line structured light patterns projected by a projector, and a conicoid method is used to improve the accuracy. The relative error of this method is 0.26%. The relative error of our method is 0.03%. From the above comparison we can see that the accuracy of the method in this paper is close to that of the method in [1], but much higher than the method in [25]. However, the method in [1] needs to post artificial markers on the surface of the object to expand the measurement range, the operation process is complicated. In comparison, our proposed method is easier to operate.

## 5. Conclusions

In this study, a free-moving surface reconstruction technique using binocular structured light was proposed. To achieve a better practical effect and ensure reconstruction accuracy, an improved scheme was proposed for system positioning and posture optimization and an extended orthogonal iteration algorithm was designed for this system. Compared with the traditional orthogonal iteration algorithm, this algorithm fully utilizes the binocular matching information making it more suitable for binocular positioning system. Furthermore, to solve the problem of error accumulation in the push-broom process, a global optimization method was proposed and the simulation results showed that the extended binocular orthogonal iteration algorithm improves the accuracy of pose estimation between adjacent frames. Moreover the global optimization method unifies the poses of the system of each frame in the world coordinate system, and effectively avoids the accumulation of drift errors in the push-broom process. Physical experiments for reconstructing a free-form surface and a flat calibration board were designed to verify the accuracy of the proposed scheme. Experimental results showed that when the system is 1.5 m away from the surface to be measured, the RMS error is reduced from 0.9 mm to 0.4 mm. This shows that the global optimization algorithm can effectively restrain the error of pose calculation, and the proposed scheme has the ability to precisely reconstruct the surface of objects.

## Figures and Tables

**Table 1:** The pre-calibrated variables and the variables to be determined.

Pre-Calibrated Parameters		Unknown Parameters	
Item	Parameters	Item	Parameters
Extrinsic parameters of the Binocular camera	Rotation matrix	The pose of the camera in the world coordinate system.	Rotation matrix
	Translation vector		Translation vector
Intrinsic parameters of the left camera	Focal length	The coordinates of the light stripes in the camera coordinate system	*X*-axis coordinates
	Principal point coordinate		*Y*-axis coordinates
	Distortion coefficient		*Z*-axis coordinates
Intrinsic parameters of the left camera	Focal length	The coordinates of the light stripes in the word coordinate system	*X*-axis coordinates
	Principal point coordinate		*Y*-axis coordinates
	Distortion coefficient		*Z*-axis coordinates

**Table 2:** Camera parameters.

Camera	f_u_/Pixels	f_v_/Pixels	u_0_/Pixels	v_0_/Pixels	k_c_
Left	2564.60	2464.09	635.77	509.03	[−0.17, 0.18, 0.003, 0.0004, 0.00]
Right	2571.32	2570.61	625.87	503.24	[−0.13, 0.16, 0.001, 0.0003, 0.00]
om	[−0.01615, 0.15960, 0.01905]				
T_0_/mm	[−215.2829, −2.1204, 16.5770]				

**Table 3:** Error from 3D points to the fitted plane.

Error Type	Maximum Error	Average Error	RMS Error	
Before optimization	2.32 mm	0.67 mm		0.9052 mm
After optimization	1.51 mm	0.43 mm		0.4177 mm
